# Influence of Matrix and Surfactant on Piezoelectric and Dielectric Properties of Screen-Printed BaTiO_3_/PVDF Composites

**DOI:** 10.3390/polym13132166

**Published:** 2021-06-30

**Authors:** Carlo Carbone, Mohammed Benwadih, Giulia D’Ambrogio, Minh-Quyen LE, Jean-Fabien Capsal, Pierre-Jean Cottinet

**Affiliations:** 1 Université Grenoble Alpes, CEA-Liten, 17 Avenue des Martyrs, 38000 Grenoble, France; mohammed.benwadih@cea.fr; 2 University of Lyon, INSA-Lyon, LGEF, EA682, 69621 Villeurbanne, France; giulia.dambrogio@insa-lyon.fr (G.D.); minh-quyen.le@insa-lyon.fr (M.-Q.L.); jean-fabien.capsal@insa-lyon.fr (J.-F.C.); pierre-jean.cottinet@insa-lyon.fr (P.-J.C.)

**Keywords:** piezoelectric, dielectric, screen-printing, composite, fluorinated surfactant, surface functionalization, characterization, polarization

## Abstract

The aim of this paper was to provide insight into the impact of matrix and surfactants on the rheology, morphology, and dielectric and piezoelectric properties of screen-printed BaTiO_3_/PVDF composites. Two matrices were compared (PVDF–HFP and PVDF–TrFE), and lead-free BaTiO_3_ microparticles were added in volume fractions of 30% and 60%. Here, we demonstrated that the presence of surfactants, helping to prevent phase separation, was crucial for achieving a decent screen-printing process. Fourier-transform infrared (FTIR) spectroscopy together with scanning electron microscopy (SEM) showed that the two “fluoro-benzoic acid” surfactants established stable bonds with BaTiO_3_ and improved the dispersion homogeneity, while the “fluoro-silane” proved to be ineffective due to it evaporating during the functionalization process. PVDF–TrFE composites featured a more homogeneous composite layer, with fewer flaws and lower roughness, as compared with PVDF–HFP composites, and their inks were characterized by a higher viscosity. The samples were polarized in either AC or DC mode, at two different temperatures (25 °C and 80 °C). The 30% BaTiO_3_ PVDF–TrFE composites with two fluorinated surfactants featured a higher value of permittivity. The choice of the surfactant did not affect the permittivity of the PVDF–HFP composites. Concerning the *d*_33_ piezoelectric coefficient, experimental results pointed out that PVDF–TrFE matrices made it possible to obtain higher values, and that the best results were achieved in the absence of surfactants (or by employing the fluoro-silane). For instance, in the composites with 60% BaTiO_3_ and polarized at 80 °C, a *d*_33_ of 7–8 pC/N was measured, which is higher than the values reported in the literature.

## 1. Introduction

Piezoelectric composites made of a polymeric matrix and inorganic fillers have been the object of much research due to them being cost-effective, readily processable, and mechanically flexible, with properties that can be easily tuned [1,2,3,4]. Piezoelectric composites can be employed in sensors, actuators, voltage generators, resonant systems [5], microelectronics [5,6], the aerospace industry [7,8], civil engineering [7,9,10,11], the nuclear industry [5], and the medical field [12].

Lead-based materials such as PZT (lead zirconate titanate), PMN (lead magnesium niobate), and PZN (lead zinc niobate) have been widely used for several years as ceramic fillers in piezoelectric composites because of their excellent performance in terms of piezoelectricity and thanks to their high-temperature stability [13]. In order to avoid any toxicity problems and environmental concerns, lead-free fillers tend to capture the interest of researchers: BaTiO_3_ ceramic particles have been considered to be some of the most favorable alternatives to lead-based ceramics [5]. BaTiO_3_ exhibits a Curie temperature of ~120 °C [13] and a piezoelectric coefficient (*d*_33_) of about 331 pC/N [14]; these values are lower than those of their lead-based counterparts, thus limiting the use of BaTiO_3_ in applications of piezoelectric composites. Great efforts have been made by many researchers to enhance both the temperature and the piezoelectric properties for this material [15,16].

Moreover, the polymeric matrix plays a crucial role in composite properties such as the dielectric permittivity, the electrical conductivity, and the mechanical flexibility. The first objective of this paper consisted of evaluating the influence of the polymer matrix on the dielectric and piezoelectric properties of a composite filled with lead-free BaTiO_3_ particles. Actually, the choice of the matrix significantly contributes to the efficiency of the poling process; the electrical conductivity of the matrix should be as high as possible. In such a case, at a fixed poling field, a higher *d*_33_ is observed [17]. Another parameter that must be taken into account is the permittivity of the matrix; a higher permittivity results in a more uniform penetration of the electric field within the material [18], resulting in an improved poling process. In the case of an AC field, the determining factors are the frequency and the amplitude of the poling field. In general, an oscillating electric field leads to a less effective polarization, since the dipoles may not follow the direction of the field itself; the optimal poling frequency is the one that minimizes the dielectric loss tangent. In this study, two types of PVDF-based polymers were investigated: PVDF–HFP (non-ferroelectric) and PVDF–TrFE (ferroelectric). The reason we chose the PVDF matrix was because of its interesting properties, including a large frequency bandwidth, high sensitivity, excellent robustness, easy processing, high environmental and chemical stability, and reliability [19].

Another key issue of this work was the homogeneity of the filler dispersion within the matrix, which was, to some extent, influenced by the wettability between the filler itself and the polymeric solution. Filler agglomeration may cause an increase in the number of stress concentration points, leakage paths, and dielectric losses, with a disruption of the electric field uniformity. At the interface between the liquid polymeric matrix and the ceramic particles, the interfacial free energy (γ_SL_) affects the wettability between the two phases [20,21]. A minimization of “γ_SL_” leads to an increased wettability and, thus, improved homogeneity of the particle dispersion. 

Several solutions have been proposed in the literature, such as the introduction of surfactants or coupling agents into the composites, making it possible to optimize their electromechanical coupling coefficient. For instance, silanes tend to enhance the mechanical resistance and elastic modulus [22]. Alternatively, dopamine hydrochloride increases the breakdown field, the permittivity, and the energy storage capability in PVDF/BaTiO_3_ composites [23]. Furthermore, IDPI (an epoxy-functionalized coupling agent) speeds up the poling process, improving *d*_33_ (at a fixed time) in PZT/epoxy composites [24]. Furthermore, KH550 (a silane), if added in 2% to BTO/PVDF composites, maximizes the breakdown field but deteriorates the dielectric behavior [25]. Lastly, SCA (a silane), dissolved in ethanol, simultaneously increases the piezoelectric coefficient and the stiffness of PVDF–TrFE/BTO composites [26]. 

One of the aims of this paper was to assess the influence of fluorinated surfactants on the piezoelectric and dielectric properties of BaTiO_3_/PVDF composites, as well as on the rheology and the homogeneity of the filler dispersion. We demonstrated that the presence of surfactants, preventing phase separation between the polymer and its solvent, was crucial for achieving a decent screen-printing process. 

Screen-printing is a promising technique to be employed in an industrial context, as it is fast, efficient, simple, robust, cheap, flexible, and not much studied as of yet. It allows the deposition of multiple layers such as a capacitor with a “sandwich” structure. Its throughput is in the range of 2–3 m^2^/s, which is lower than that of gravure printing (3–60 m^2^/s), but higher than for ink-jet (0.01–0.5 m^2^/s) and slot-die (~0.2 m^2^/s) printing [27]. Another reason why screen-printing should be chosen is because it guarantees a huge range of thicknesses (1–20 µm), comparable to that of ink-jet printing (0.01–20 µm), but much broader than for gravure (0.1–1 µm) and slot-die (0.1–2 µm) printing [27]; an increase in the thickness of a piezoelectric composite leads to an improvement of the piezoelectric and dielectric properties [28]. The thickness is directly correlated with the viscosity of the ink, whose range should be within the interval 0.5–50 Pa·s, in order to comply with the screen-printing technique [27]. One of the challenges (still open) is to produce composite layers with reduced roughness and porosity. To reach this goal, the idea here was to improve the printing process and efficiency by reducing solvent consumption and ink waste. One of the purposes of this experimental work was to optimize the screen-printing process.

The research goals of this experimental work can be summarized as follows:

- Investigate the impact of the matrix and surfactants on the rheology, morphology, and dielectric and piezoelectric properties of BaTiO_3_/PVDF composites.
- Determine the effectiveness of fluorinated surfactants, and how the functionalization of BaTiO_3_ affects the rheology, morphology, and dielectric and piezoelectric properties of BaTiO_3_/PVDF composites.
- Improve the screen-printing process, in order to obtain smooth, homogeneous, and flawless dielectric layers: this was done by avoiding phase separation and by understanding the most suitable viscosity value of the inks.
- Optimize the poling process parameter, in order to obtain the best piezoelectric performance.

## 2. Material Fabrication

### 2.1. Material Selection

Sigma Aldrich^®^ (Darmstadt, Germany) BaTiO_3_ powder was used (ρ = 6.02 g/cm^3^, M_w_ = 233.19 g/mol), with a maximum dimension of 3 µm (99%) and an average size (diameter) of less than 1 µm.

Here, we focused on two typical PVDF copolymers: 

(1) PVDF–TrFE (polyvinylidene fluoride–cotrifluoroethylene): a ferroelectric polymer with a Curie temperature (T_c_) of 80–140 °C and a piezoelectric charge coefficient *d*_33_ = 25–40 pC/N. Both T_c_ and *d*_33_ values are affected by the proportion between TrFE and VDF monomers [5,29].

(2) PVDF–HFP (polyvinylidene fluoride–cohexafluoropropylene): a non-ferroelectric polymer, weakly piezoelectric, with a *d*_33_ of about 3 pC/N [30]. The permittivity and conductivity of PVDF–TrFE are lower than for PVDF–HFP [31,32], provoking an inferior electric field transmission within the material. As a consequence, there is a greater loss of effectiveness during the poling process, resulting in a depletion of the dielectric and piezoelectric properties of the material itself.

Both PVDF–HFP and PVDF–TrFE were purchased from Sigma Aldrich^®^ (Darmstadt, Germany). The technical information of these two polymeric matrices is described in Table 1.

Table 2 summarizes the physical and chemical properties of the three surfactants, identified by their short names “3F-ben”, “3F-met”, and “3Si”. The designation “None” refers to a formulation without surfactant. Each surfactant was provided by Sigma Aldrich^®^ (Darmstadt, Germany).

To make the conductive electrodes of each sample, a conductive polymer PEDOT (poly(3,4-ethylenedioxythiophene)–poly(styrene-sulfonate)) (Clevios S V4, Heraeus Deutschland GmbH & Co, Leverkusen, Germany) was used, dispersed in H_2_O with some additives: 2,2’-oxydiethanol (13%), benzensulfonic acid/2,3- dihydrothieno[3,4-b]-1,4-dioxin (1%), and polyurethane (4%).

The metallic contacts with the sample were realized by depositing a conductive Ag-paste (EMS CI-1001, Nagase Engineered Materials Systems Inc., Delaware, OH, USA), made of silver particles (61%), vinyl monomers, and methyl ethyl ketone (MEK) solvent. The Ag-paste is characterized by an electrical resistance <0.015 Ω.

### 2.2. Material Preparation

#### 2.2.1. BaTiO_3_ Surface Functionalization

For the preparation of the functionalized particles, 5 g of BaTiO_3_ and 0.3 g of surfactant were diluted in 80 mL of ethanol absolute anhydrous (99.99%). This proportion was established in order to obtain nearly 5–6% weight fraction of surfactant.

With the aim of breaking up the particle aggregates, the mixture was subjected to ultrasonication for 5 min, and then agitated by magnetic stirring with a rotation speed of 1000 rpm. In order to get the surfactant to successfully form a molecular shell around the BaTiO_3_ particles, the stirring process lasted for 1.25 h, which was not excessively long so as to avoid the creation of a thick surfactant shell that may deplete the effectiveness of functionalization. 

Afterward, the suspension was poured in six vials and was centrifuged at 4500 rpm (in a VWR CompactStar^®^ CS 4 centrifuge, Radnor, PA, USA) for 5 min, enabling the separation of the solid powder from the ethanol. The process was repeated three times, rinsing every vial twice with virgin ethanol. The remaining solid powder was then placed in a Memmert^®^ oven (Schwabach, Germany) at 70 °C for 3 h, in order to completely evaporate the residual ethanol. 

#### 2.2.2. BaTiO_3_/PVDF Mixing

The polymers (PVDF–HFP or PVDF–TrFE) and Sigma Aldrich^®^ (Darmstadt, Germany) TEP (M_w_ = 182.15 g/mol, ρ = 1.072 g/mL, T_b_ = 220 °C) were respectively mixed with the weight ratio of 20%–80%, using a “RZR 2020” (Heidolph, Schwabach, Germany) mixer (with a glass helix). Mixing occurred under magnetic stirring (rotation speed: 1600 rpm) for 9 h at T = 100 °C. The polymeric solutions were stocked in hermetically closed vials to prevent solvent evaporation.

Each group (3F-ben, 3F-met, 3Si, None) of BaTiO_3_ particles was mixed with each polymer (PVDF–HFP and PVDF–TrFE), in 30% volume fraction of BaTiO_3_ particles. The solvent volume, i.e., the volume evaporated after the deposition process, was not considered. The surfactant volume could be neglected as well. In order to assess the effect of the filler concentration, we also prepared samples with a higher volume fraction of 60%. For the sake of simplicity, only functionalized “3F-met” and “3Si” particles, blended with the PVDF–TrFE polymer matrix, were used for these samples. The mixing process occurred at 1500 rpm (Heidolph, RZR 2020 mixer, Schwabach, Germany) for 1.25 h.

#### 2.2.3. Screen-Printing and Solvent Evaporation

**Figure 1:**
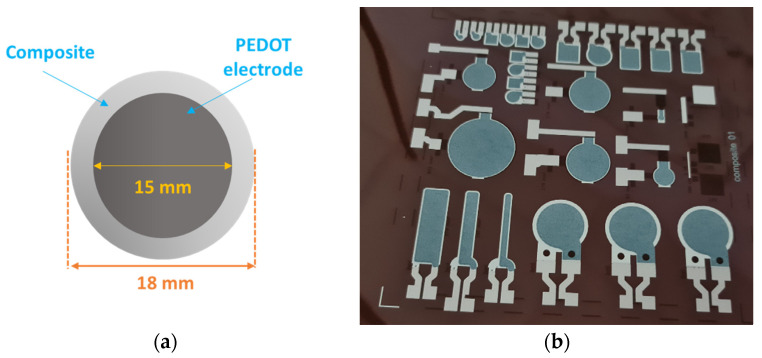
(**a**) Upper view of the circular capacitor; (**b**) real photo of the printed samples with different shapes.

After preparing the composite blend, we opted for an EKRA^®^ X5-STS (Global BizTeK Co., Gyeonggido, South Korea) screen-printer to make a series of circular capacitors. Figure 1a presents the typical sandwich structure of a sample, which was printed on a 65 μm thick polyimide substrate. In order to remove fingerprints and impurities, the substrate was washed with acetone and propanol, and was then exposed to a UV lamp for 1 min. Figure 1b shows the real samples, which were screen-printed on the polyimide foil.

The screen-printing mask was a square shape with a side of 560 mm, and it had a texture of intertwined wires forming an angle of 22.5°. A photo-crosslinked resin (FL260-EOM) was used to define a certain pattern on the mask itself. The masks employed for the PEDOT and Ag-paste inks consisted of stainless-steel wires (each mask was characterized by a different value for the wire diameter). For each BaTiO_3_/PVDF ink, a mask with polyester wires was used. All the squeegees were made of the same elastomer (polyurethane SERILOR^®^), characterized by a hardness of 75 SH and a length of 18.5 cm. 

Some key parameters were set in the screen-printing equipment such as squeegee speed (30 mm/s), squeegee force (30 N), and snap-off (i.e., the distance between the mask and the substrate, equal to 2.2 mm).

Before printing, the PEDOT was blended for at least 20 min via a metallic helix of the RZR 2020 (Heidolph, Schwabach, Germany) mixer that was set at a rotational speed of 3000 rpm. The Ag-paste was mixed using a spatula, for a couple of minutes, until decent homogenization was reached. Some attention had to be paid to each piezoelectric ink. Screen-printing had to be done at least 1 day after ink production; otherwise, segregation of the solvent would occur, with subsequent alteration of viscosity and homogeneity.

**Figure 2:**
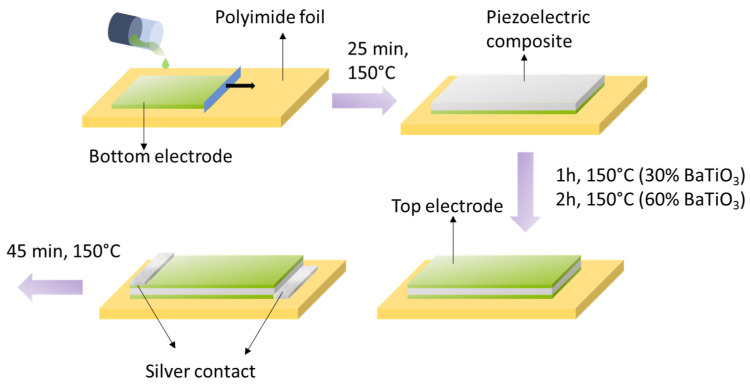
Scheme of the deposition process.

To carry out solvent evaporation, each deposited layer was subjected to a thermal treatment by means of a heating plate. The time and temperature of the treatment varied depending on the thickness of the deposited layer and of the solvent. Figure 2 summarizes the whole printing procedure. 

For the 30% BaTiO_3_ composites, two deposition steps were carried out, while one more step was needed (three in total) for the 60% counterpart. Between each deposition step (called mid-step), a partial solvent evaporation was performed for 5 min at 150 °C. For the top-electrode, a double-step deposition was also done, with a longer evaporation process than for the bottom electrode. 

#### 2.2.4. Poling

**Figure 3:**
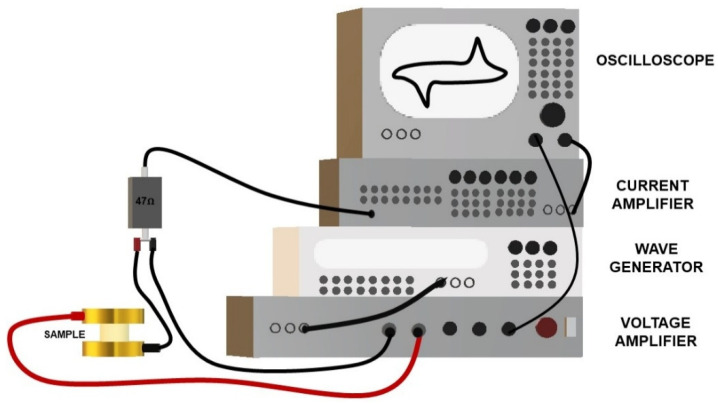
Scheme of the poling experimental setup.

Figure 3 illustrates the experimental setup of the poling process. A waveform generator (Agilent 33210A, Keysight Technologies Inc., Santa Rosa, CA, USA) was used to choose AC or DC mode, and it was coupled with an amplifier (10/10 B-HS, TREK Inc., Novi, MI, USA) in order to enhance the input signal by a factor of 1000. The resulting current was detected via a low-noise current preamplifier (SR570, Stanford Research Systems Inc., Sunnyvale, CA, USA), by choosing a suitable sensitivity. It was fundamental to avoid any undesired distortion of the “current–voltage” hysteresis loop (a yardstick for a qualitative assessment of the poling process). To create the electrical contact, the sample was clamped between two copper electrodes to which was applied the input voltage. The composites with 30% BaTiO_3_ were poled at 25 °C, under the AC current, with a frequency of 7 Hz and a peak-to-peak electric field of 10 kV/mm, for 20 min. Some samples were tested with a peak-to-peak field of 20 kV/mm, and no dielectric breakdown occurred, while all the samples faced breakdown if poled with an electric field of 25 kV/mm. As reported in [33], an electric field of 5 kV/mm (applied for 30 min) makes it possible to obtain good piezoelectric performances. The applied poling field should be higher than the coercive field, which, for BaTiO_3_/PVDF composites with 30% BaTiO_3_, is about 2–2.5 kV/mm [34]. Consequently, it appeared to be inappropriate to use a poling field of 20 kV/mm, since this was close to the breakdown value. It was, thus, preferable to carry out polarization at a value of 10 kV/mm. Conversely, the polarization of the samples with 60% BaTiO_3_ was performed via DC current, for 20 min, at 25 °C and at 80 °C, with a poling field of 10 kV/mm. Even more so in this case, this value was preferred as opposed to a higher poling field such as 20 kV/mm, in order to minimize the risk of dielectric breakdown. In fact, the probability of breakdown tended to increase at larger particle concentrations [35] and film thicknesses [36]. For a polarization at high temperature, the sample was placed in an oven (Vötsch^®^ VT-7004, CMR AS, Bergen, Germany).

## 3. Characterization Method

### 3.1. Fourier-Transform Infrared (FTIR) Spectroscopy

FTIR spectroscopy, carried out by means of a specific equipment (VERTEX 70, BRUKER, Billerica, MA, USA), was a suitable technique to show the effectiveness of the functionalization of BaTiO_3_ particles. Each BaTiO_3_ powder, characterized by a different formulation (“3F-ben”, “3F-met”, “3Si”, “None”), was encapsulated into potassium bromide (KBr) pellets (transparent to FTIR). The drawback of KBr pellets is that their moisture content leads to OH band distortion in the spectra.

### 3.2. Rheometric Analysis

In order to perform rheological characterization, a rotational rheometer (MCR 300, Anton Paar France, Les Ulis, France), featuring a Peltier heating plate and a CP50-1 rheometric head (cup and cone geometry), was employed. Measurements were carried out at room temperature (20 °C) for 100 s, to acquire 41 data points. The rotational speed was set to ramp up at a progressively increasing rate from 1 s^−1^ to 100 s^−1^, and then ramp down from 100 s^−1^ to 1 s^−1^. To improve accuracy, each sample was tested three times and an average value was calculated.

### 3.3. Profilometry

A FP10 profilometer (Toho Technologies, Chicago, IL, USA) was used to measure the thickness and the roughness of each deposited layer. Some key parameters were set, including the scan length (5000 μm), scan speed (60 μm/s), sampling rate (100 Hz), and stylus force (3 mg).

### 3.4. Scanning Electron Microscopy (SEM)

With the aim of assessing the homogeneity of dispersion of BaTiO_3_ particles within the polymer matrix, imaging analysis of the composite’s cross section was carried out by means of a GeminiSEM 460 device (Zeiss, Jena, Germany). The samples were cryofractured in liquid nitrogen to obtain a sharp cut of the material. Then, each sample was subjected to PVD sputtering (physical vapor deposition), by means of an EMSCOPE SC500 device (Emzer, Barcelona, Spain) so as to be covered by a 10 nm thick layer of platinum, a necessary step to make them conductive. The process lasted 30 s and occurred at a pressure of 0.03 torr (~4 Pa) and a current of 16 mA.

### 3.5. Broadband Spectroscopy

Dielectric measurements were performed via a frequency response analyzer (SI-1255, Solartron, Oak Ridge, TN, USA). The equipment was employed to analyze the frequency dependence of both the real and the imaginary part of the permittivity ($\varepsilon_{33}^{\prime }$). The following parameters were set: AC mode with a voltage amplitude of 1 V, frequency logarithmic ramp from 1 Hz to 1 MHz, 31 data points acquired during 3 min at room temperature.

### 3.6. Piezoelectric Characterization

The measurement of the piezoelectric charge coefficient (*d*_33_) was performed by means of a specially designed setup with high sensitivity, composed of the following:

- a dynamic oscillator (PI 246-50) coupled with a waveform generator (33522B, Keysight Technologies Inc., Santa Rosa, CA, USA) together with a voltage amplifier (Model 20/20C, TREK Inc., Novi, MI, USA), making it possible to generate a sinusoidal force with a tunable amplitude and frequency,
- a C11 force sensor (HBM, Darmstadt, Germany),
- a charge sensor (KISTLER, Type 5015, Winterthur, Switzerland), connected to the sample by means of copper electrodes.

The measurements were performed at a frequency of 1 Hz, with a force amplitude of about 700 N and a bias of 70–100 N. Finally, real-time signals were simultaneously recorded using DEWE platform (Sirius, 8XSGT, SI-1420 Trbovlje, Slovenia). 

Here, “*C_PP_*” and “*F_PP_*” are respectively the peak-to-peak charge and force amplitudes, “*A_C_*” is the area on which the charge was accumulated (i.e., the area of the 15 mm circular PEDOT electrode), and “*A_F_*” is the area on which the force was applied (i.e., the total area of the capacitor with 18 mm diameter). The piezoelectric charge coefficient (*d*_33_) was determined according to the following equation:

(1) d33=CPPACFPPAF.

## 4. Results and Discussion

This section is outlined as follows:

(1) FTIR spectra of BaTiO_3_ functionalized particles: detecting the presence of each surfactant on BaTiO_3_ particles to better understand the mechanism of functionalization;
(2) Rheological characterization: evaluating viscosity curves as a function of the strain rate of each ink, to determine how the formulation affected the rheology;
(3) Profilometry: determining the average thickness and the roughness of each printed composite;
(4) SEM cross-section micrographs: assessing the homogeneity of filler dispersion within the matrix of each composite, i.e., characterized by a different formulation and a different filler concentration;
(5) Broadband dielectric properties: analyzing the frequency dependence of the permittivity and tangent loss;
(6) Piezoelectric properties: evaluating the piezoelectric charge coefficient (*d*_33_) of all composites.

### 4.1. FTIR Spectra of BaTiO_3_ Functionalized Particles

**Figure 4:**
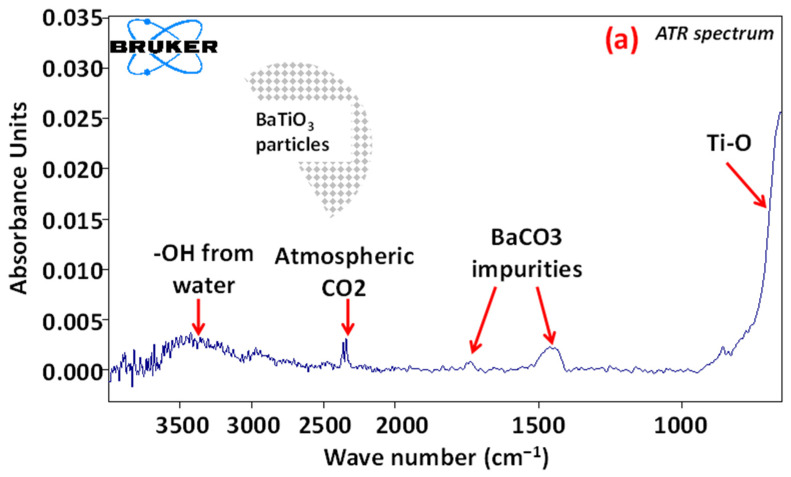

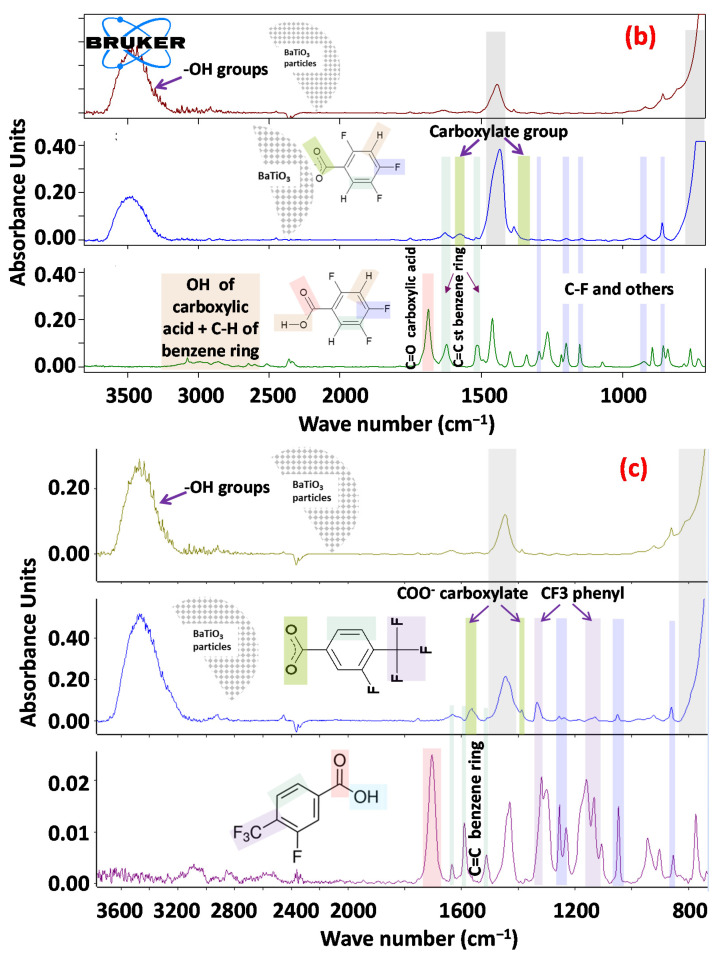

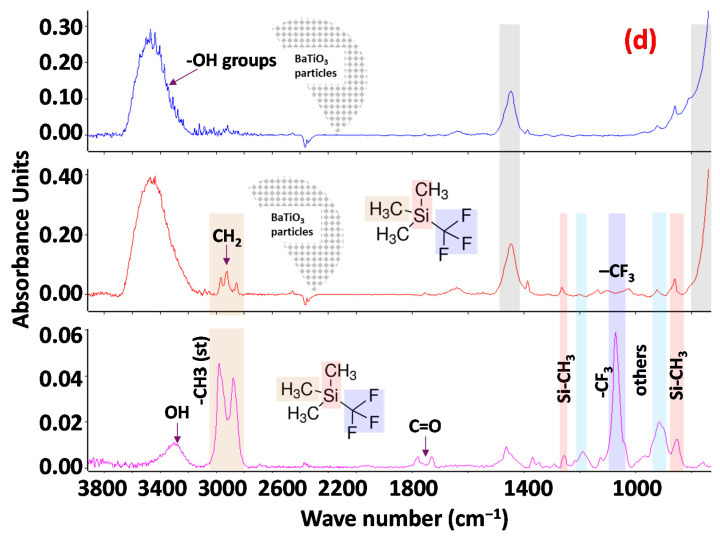
FTIR spectra of BaTiO_3_ particles: (**a**) “None”; (**b**) “3F-ben”; (**c**) “3F-met”; (**d**) “3Si”.

Figure 4 compares the spectra of BaTiO_3_ functionalized particles (“3F-ben”, “3F-met”, “3Si” surfactants) with that of the unfunctionalized case (“None”). As shown in Figure 4a, the surface of BaTiO_3_ unfunctionalized particles appeared to be spontaneously covered by –OH groups that probably stemmed from the atmospheric humidity. Moreover, the analysis led to the detection of certain BaCO_3_ impurities, which were inevitable during the industrial refining process of the powder.

Figure 4b displays the FTIR spectrum of BaTiO_3_ particles treated with surfactant “3F-ben”. The natural presence of –OH groups on the BaTiO_3_ surface turned out to be an advantage, since the carboxylic group of surfactant “3F-ben” was able to form stable bonds with the BaTiO_3_ particles. This can be proven by simply noticing that the carbonyl group (–C=O) of the surfactant “3F-ben” (localized around k = 1700 cm^−1^) disappeared to form a carboxylate group (–COO^−^). This creation established strong interactions with the surface of the filler, which was stabilized by resonance (mesomeric effect) caused by delocalization of the negative charge all along the carboxylate ion. Such a phenomenon is clearly identified by the presence of two peaks in the FTIR spectrum of Figure 4b (at around k = 1500 cm^−1^).

Similar considerations can be made for surfactant “3F-met”, which seemed to have strong interactions with the –OH group of BaTiO_3_. Some significant peaks, associated with the –CF_3_ group at k = 1100 cm^−1^ and 1300 cm^−1^, can be seen in Figure 4c. Different scenarios were observed for the surfactant “3Si” as illustrated in Figure 4d, whereby the CF_3_ and CH_3_ groups disappeared; the latter was believed to transform into CH_2_ since it did not strongly interact with –OH. This result suggested that the surfactant evaporated or degraded during ethanol evaporation at 70 °C, which was higher than the boiling point of the surfactant itself.

### 4.2. Rheological Properties

**Figure 5:**
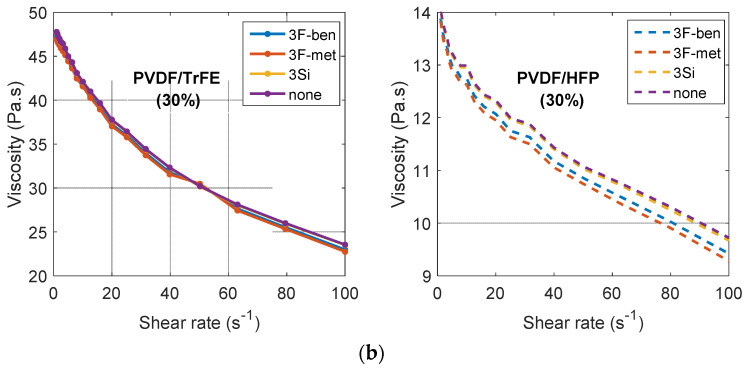

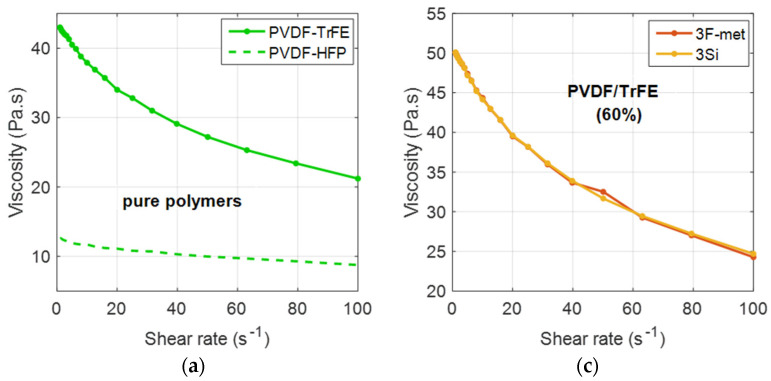
Viscosity of different solutions as a function of shear rate: (**a**) PVDF–HFP and PVDF–TrFE polymers; (**b**) composites with 30% BaTiO_3_ in PVDF–TrFE or PVDF–HFP; (**c**) 60% BaTiO_3_ in PVDF–TrFE (with surfactants “3F-met” and “3Si”).

Firstly, the viscosity of each polymeric solution was measured as a function of the shear rate. As displayed in Figure 5a, the viscosity of PVDF–TrFE was revealed to be higher than that of PVDF–HFP, which may be due to the fact that the PVDF–TrFE polymeric chains, since they were more polar, tended to have stronger interactions with each other, giving more resistance to flow. Another reason could possibly have originated from the mixing process at 100 °C between the TEP solvent and each polymer. In fact, the PVDF–HFP/TEP solution led to greater solvent evaporation than PVDF–TrFE/TEP. Thanks to its weaker interactions with the solvent molecules, the PVDF–HFP tended to let the TEP “escape” more easily in the vapor phase. As expected, both polymeric solutions exhibited a pseudo-plastic behavior, where the viscosity decreased with an increased shear rate. This effect was more obvious in the case of PVDF–TrFE, where the decrease was clearly more abrupt.

Figure 5b describes the viscosity versus the shear rate. Tests were performed on both PVDF–HFP/BaTiO_3_ and PVDF–TrFE/BaTiO_3_ inks with the same volume concentration of 30%, and with different surfactants (3F-ben, 3F-met, 3Si), as well as the formulation without surfactant (None). In each case, the viscosity values were within the ideal range imposed by the screen-printing process (0.5–50 Pa·s) [27], even at low shear rate. Regardless of the ink composition, the ramp-up curve was found to almost overlap with the ramp-down curve, suggesting that the thixotropic behavior was not very remarkable. As expected, PVDF–TrFE/BaTiO_3_ exhibited a higher viscosity than PVDF–HFP, which correlated perfectly with the previous results of Figure 5a. 

In the case of the PVDF-TrFE/BaTiO_3_ composite, the surfactants “3F-ben” and “3F-met” led to a slightly decreased viscosity with respect to the “3Si” and “None” formulations, reflecting a better transmission of shear stress from the matrix to the particle. Nevertheless, such an effect was not significant, confirming that the surfactant did not have any influence on the rheological properties of the composite. This conclusion was even more convincing in the case of the PVDF–HFP/BaTiO_3_ inks, where no difference in viscosity was observed, regardless of the different surfactants used.

To better assess the influence of the particles’ concentration on the rheological properties, a similar test was carried out on the PVDF–TrFE composites filled with 60 vol.% BaTiO_3_ and with two different surfactants: “3F-met” and “3Si”. The results of Figure 5 allowed us to conclude that a higher particle content led to higher observed viscosity. It is illustrated in Figure 5c that, at low shear rate, the viscosity slightly exceeded the ideal limit value of 50 Pa·s [27]. This would not be a main issue because screen-printing likely occurs at a higher strain rate. Again, the viscosity of the ink with surfactant “3Si” was revealed to be higher than the one with surfactant “3F-met”. To some extent, regardless of the particle concentration (30% or 60%), the surfactant had very little effect on the rheology of the samples. On the contrary, the polymer matrix, as well as the particle content, was found to substantially modify the viscosity.

### 4.3. Profilometry Analysis 

Table 3 depicts the average roughness (R_a_) and the average thickness (t_a_) of each composite layer. The results highlighted that R_a_ and t_a_ did not seem to be considerably affected by the surfactant. Conversely, the PVDF–TrFE composites exhibited much lower roughness with respect to the PVDF–HFP, regardless of which surfactant was chosen. This may be due to the fact that PVDF–TrFE was characterized by better compatibility between the substrate and the composite layer, as well as between the particles and the matrix. The composite layers with 60% BaTiO_3_ turned out to be considerably thicker than the ones with 30% because of their higher-viscosity inks and their three-step deposition process (as opposed to two steps in the case of 30%). The uncertainty with regard to the thickness of the samples was estimated to be between 10% and 20%, which means that the thickness value was very inhomogeneous within the dielectric layer (there was a certain amount of waviness). This implies some uncertainty in the value of the applied electric field during the poling process, as well as in the value of the permittivity.

### 4.4. SEM Cross-Section Images

**Figure 6:**
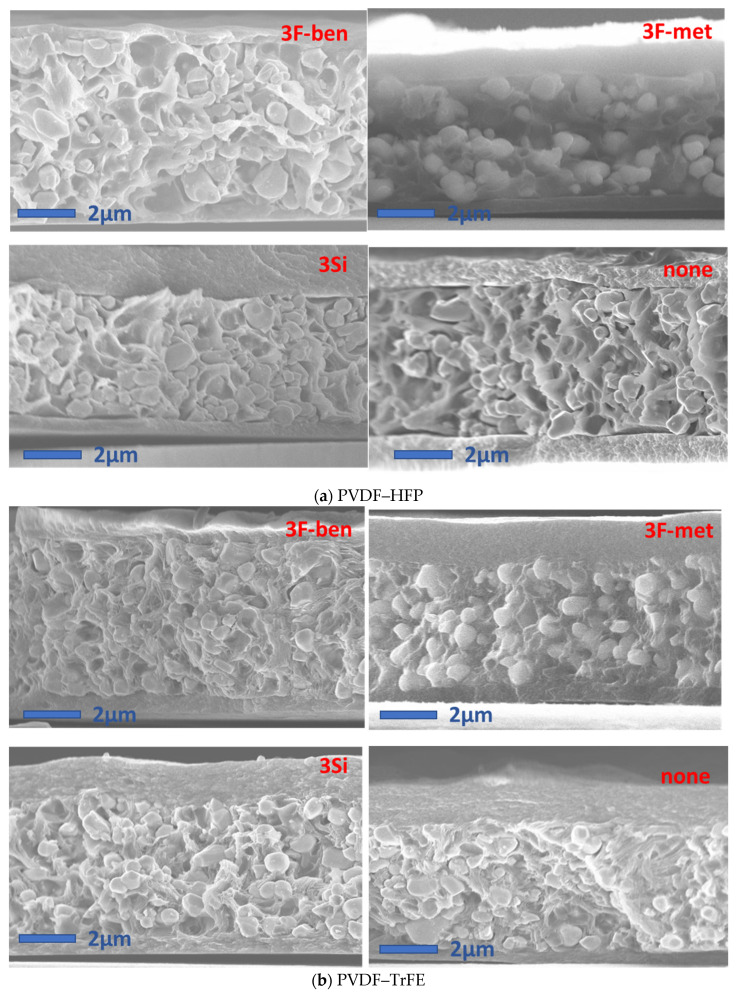
SEM micrographs of composites with (**a**) 30% BaTiO_3_ in PVDF–HFP, and (**b**) 30% BaTiO_3_ in PVDF–TrFE.

Figure 6 displays the SEM micrographs of the cross-sections of the eight samples with 30% filler, of which the matrix was a combination of two types of polymers (PVDF–HFP, PVDF–TrFE), and four types of functionalizations (3F-ben, 3F-met, 3Si, none). In some images, e.g., in the ones with PVDF–HFP (3F-met) and PVDF–TrFE (3F-ben, 3F-met), some parallel surfaces were visible at the bottom and/or at the top, representing the PEDOT electrodes. Firstly, it should be pointed out that particles in all PVDF–HFP composites appeared to be clearly de-bonded from the matrix, reflecting an unstable interface between the polymer and the BaTiO_3_ powder. In other words, regardless of the surfactant, compatibilization between these two phases could not be assured. PVDF–HFP did not demonstrate decent interactions with either the fluorine atoms of the surfactant or the –OH groups of the BaTiO_3_ particles. 

In the PVDF–TrFE composites, surfactants “3F-ben” and “3F-met” exhibited the best results in terms of particle dispersion and homogeneity. This was due to the fact that, since TEP evaporation occurred at 150 °C, the surfactants neither degraded nor evaporated. The fact that the temperature passed the melting point of the surfactant “3F-ben” during TEP evaporation did not affect the effectiveness of functionalization at all. In the case of the surfactant “3Si”, a considerable amount of filler agglomeration was observed. This surfactant degraded/evaporated during ethanol evaporation at 70 °C, since this was higher than the boiling point of the surfactant itself. When it comes to particle dispersion, the result was similar to the case without surfactant.

**Figure 7:**
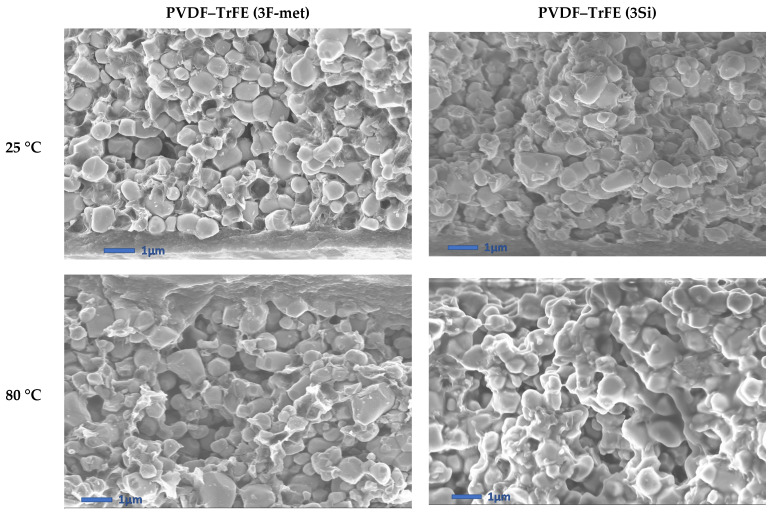
SEM micrographs of cross-sections of composites with 60% BaTiO_3_ (in PVDF–TrFE).

Figure 7 represents SEM micrographs of the cross-sections of the samples with 60% BaTiO_3_ in PVDF–TrFE, poled at two different temperatures (25 °C and 80 °C, see Section 2.2.4) and functionalized with two kinds of surfactants (“3F-met” and “3Si”). In accordance with the previous observations, the surfactant “3F-met” led to improved particle dispersion as compared with the surfactant “3Si”. Logically, a higher particle concentration would lead to a larger difference between these samples’ dispersions. In practice, however, samples with 60% BaTiO_3_ manifested the presence of several agglomerates, even with an effective surfactant (e.g., “3F-met”), meaning that it was hard to achieve a decent dispersion with a significant particle concentration. Additionally, the result highlighted that the effect of the surfactant did not seem to be affected by the poling temperature, since the SEM micrographs revealed similar results at both 80 °C and 25 °C.

### 4.5. Dielectric Properties

**Figure 8:**
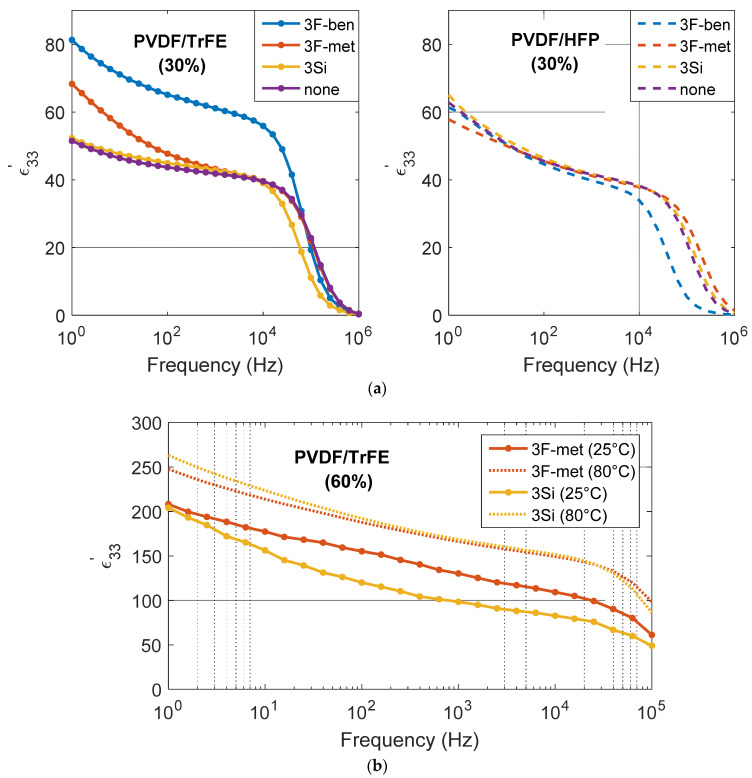
Broadband dielectric permittivity ($\varepsilon_{33}^{\prime }$) of (**a**) PVDF–HFP and PVDF–TrFE composites (30% BaTiO_3_), and (**b**) PVDF–TrFE composites (60% BaTiO_3_, surfactants “3F-met” and “3Si”).

Figure 8a depicts the real part of the dielectric permittivity ($\varepsilon_{33}^{\prime }$) of the PVDF–HFP and PVDF–TrFE composites (30 vol.% BaTiO_3_) as a function of the frequency. All samples were poled at 25 °C, under an AC current, at 7 Hz frequency and 10 kV/mm (peak-to-peak field). Regardless of the surfactant, $\varepsilon_{33}^{\prime }$(at 1 kHz) of the BaTiO_3_/PVDF composites lay within an interval of 30 and 60, which was in agreement with the empirical values found in the literature [33,37]. The first aspect that caught the eye was that the composite with the surfactant “3F-ben” and the PVDF/TrFE matrix exhibited a considerably higher value of permittivity than the other composites. This may be due to uncertainties when measuring the thickness, as $\varepsilon_{33}^{\prime }$ was estimated from measurements of the capacitance (*C_33_*), the thickness “*t*”, and the surface “*S*” according to

(2) $$\varepsilon_{33}^{\prime }=\frac{C_{33\;}\times t}{S}.$$

Since the aforementioned composite layer was not smooth, but exhibited some zones characterized by lower thickness (with respect to the one declared in input), this may have led to an overestimation of the permittivity. Except for the sample comprising PVDF–TrFE and “3F-ben”, the results of Figure 8a reveal that neither the surfactant nor the polymeric matrix had a significant impact on the relative permittivity. At this stage, the following considerations can be pointed out:

- In the case of the surfactants “3F-ben” or “3F-met”, the PVDF–TrFE matrix gave rise to an enhanced permittivity with respect to the PVDF/HFP (at *f* < 50 kHz). This behavior was further affected by the intrinsic dielectric properties of the neat polymer rather than of the surfactant itself. Actually, PVDF–TrFE has a higher value of *ε*’_33_ than PVDF–HFP [31,32]. As observed in the SEM micrographs of Figure 6, both surfactants made it possible to reach a decent particle dispersion; hence, there was no big difference between the two.
- In the case where no surfactant was used, or with the surfactant “3Si”, on the other hand, *ε*’_33_ of the PVDF–TrFE composites was lower than the corresponding value for PVDF–HFP (up to *f =* 250 Hz), while for 250 Hz < *f* < 50,000 Hz, the values were quite similar. These inconsistencies probably originated from a bad dispersion of particles inside the matrix (as proved by the SEM micrographs), which did not allow a conclusive comparison of the effect of the two matrices.
- For the same matrix (PVDF–TrFE), the surfactants “3F-ben” and “3F-met” featured higher values of *ε*’_33_ (by considering the measurement uncertainty) as compared with “3Si” and “None”. This behavior was expected considering the better homogeneity of dispersion based on the functionalization of “3F-ben” and “3F-met”, which definitively made it possible to avoid BaTiO_3_ agglomeration, in turn inducing PVDF accumulation at the composite/electrode interface.
- Generally, since the permittivity of the polymer is lower than the one of the filler [13,31,32], the transmission of electric field throughout the composite is less efficient, favoring charge accumulation at the interface between PVDF and BaTiO_3_ agglomerations. Such a phenomenon, inducing capacitance depletion caused by a lower charge accumulation at the electrodes under a given applied electric field, resulted in a lower dielectric permittivity of the whole composite.

Figure 8b depicts the real part of the relative permittivity ($\varepsilon_{33}^{\prime }$) of the PVDF–TrFE composites filled with 60% BaTiO_3_ particles and functionalized by surfactants “3Si” or “3F-met”. Each sample was poled under a DC field (10 kV/mm) and at two different poling temperatures (i.e., 25 °C and 80 °C). Logically, $\varepsilon_{33}^{\prime }$ considerably increased with the higher concentration of BaTiO_3_. No considerable difference was seen between surfactant “3Si” and “3F-met”, despite the dielectric constant for the “3Si” formulation seeming to be slightly higher. Another interesting aspect was the fact that $\varepsilon_{33}^{\prime }$ augmented with the poling temperature, which was possibly due to two effects:

- The viscosity of the matrix decreased at higher temperature; thus, particles were more prone to orient in the direction of the poling field. This behavior was observed above the glass transition temperature of the PVDF–TrFE polymer *T_G_* ~ (−35,−12) °C [38].
- The dipole orientation is a thermally activated process, where relaxation time exponentially decreases with the temperature itself (Arrhenius law [39]). In other words, the dipole orientation occurs faster under a fixed frequency. Consequently, the permittivity, inversely related to the dielectric relaxation time [39], increases.

**Figure 9:**
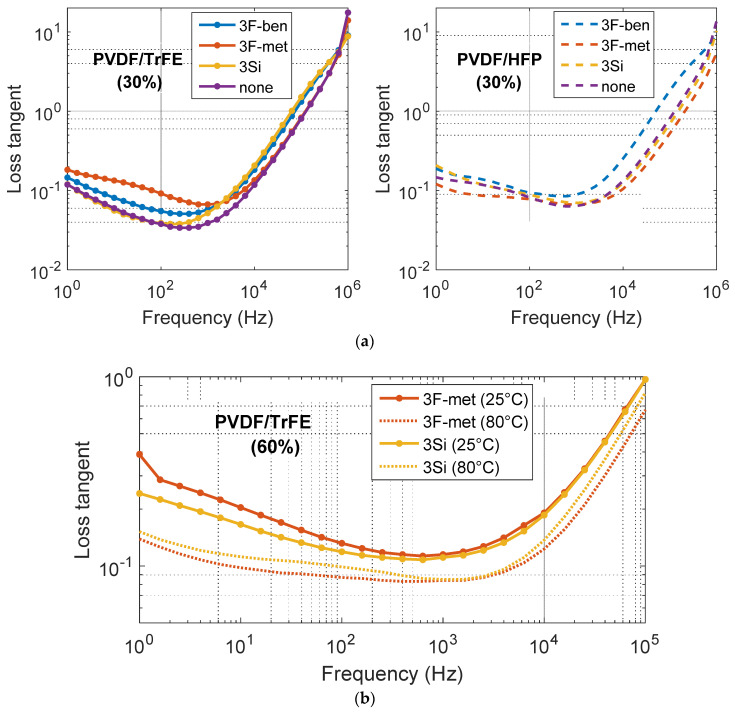
Loss tangent versus frequency of the (**a**) PVDF–HFP and PVDF–TrFE composites (30% BaTiO_3_), and (**b**) PVDF–TrFE composites (60% BaTiO_3_ + surfactants “3F-met” and “3Si”).

Figure 9a illustrates the loss tangent of the PVDF–HFP and the PVDF–TrFE composites (30 vol.% BaTiO_3_) as a function of the frequency. The values of tanδ turned out to be slightly higher than those found in the literature; at 1000 Hz, the loss tangent is usually lower than 0.05 [33,37]. This was related to the defects (which were not detected) created during the fabrication process and to the high roughness of the sample surface. This produced inclusions of the PEDOT electrode into the pits of the dielectric layer, with a subsequent increase in leakage paths and dielectric dissipation [40,41]. As a result, no consistent comparison could be made between the different formulations. Neither surfactant nor matrix seemed to significantly affect the loss tangent; its values appeared to be randomly distributed, regardless of the formulation.

Figure 9b plots the loss tangent of the same samples as a function of the frequency. On the one hand, the poling temperature substantially affected the value of tanδ, which turned out to be lower for the composites poled at 80 °C. On the other hand, the formulation did not have a significant influence. The surfactant “3Si” led to higher loss as opposed to the surfactant “3F-met” at 25 °C poling, i.e., inversely to the case of 80 °C. Such a phenomenon was perhaps caused by the random presence of defects and the roughness of each sample, impeding any relevant conclusion to be drawn. The values of tanδ were considerably increased at a higher concentration of particles, which is in line with the experimental data reported in the literature. In fact, a low percentage of dielectric dipoles will follow the orientation of the AC electric field, inducing more dielectric dissipation [42].

### 4.6. Piezoelectric Properties

Table 4 summarizes the piezoelectric charge coefficient (*d*_33_) of the eight composites filled with 30 vol.% BaTiO_3_, together with different combinations of matrix and surfactant. Li et al. showed that the *d*_33_ coefficient of the 70 vol.% BaTiO_3_/PVDF composites could reach a value of 5 pC/N [33]. Our values reported in Table 4 were considerably lower, but still in line with the literature. The main reason for this was that, in Li’s work, the volumetric fraction of BaTiO_3_ was higher (70% versus 30%), and this had a strong effect on the piezoelectric behavior of the composites [2].

**Figure 10:**
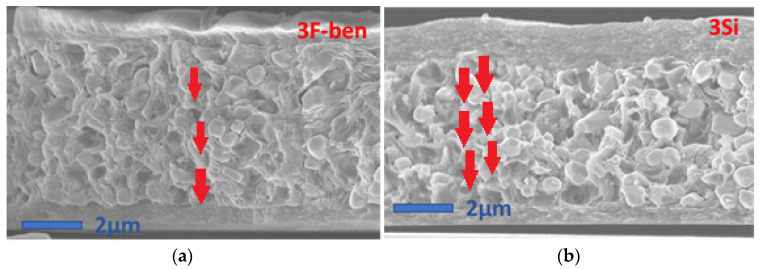
BaTiO_3_ dipole distribution within the PVDF–TrFE matrix for the formulations “3Si” (**b**) and “3F-ben” (**a**).

The results of Table 4 led to several considerations:

- Firstly, the PVDF–HFP composites featured much lower *d*_33_ values compared with their PVDF–TrFE counterparts, which was probably due to particle/matrix debonding; this implied a poor electric field penetration during the poling process and a bad mechanical stress transmission during the oscillatory tests. Another reason is related to the fact that PVDF–TrFE is a ferroelectric polymer; hence, it can better drive the electric field into the composite, improving the effectiveness of the poling process and giving rise to a higher charge accumulation at the electrodes under a mechanical solicitation.
- Secondly, the “3Si” and “None” formulations led to considerably improved *d*_33_ coefficients as compared with their “3F-ben” and “3F-met” counterparts, with approximately a twofold and fivefold increase in the case of PVDF–HFP and PVDF–TrFE, respectively. As illustrated in the SEM micrographs of Figure 10 (i.e., extracted from Figure 6), composites comprised of the surfactant “3Si” and PVDF–TrFE showed a certain amount of particle agglomeration, favoring anisotropy along the poling direction (the same was true for composites without surfactant, i.e., the “None” formulation). Furthermore, the surfactants “3F-ben” and “3F-met” established stronger interactions with BaTiO_3_, which may have hindered the orientation of dipoles at the interface between the filler particles and the matrix. Conversely, in the case of the “3Si” and “None” formulations, the dipoles were freer to rotate, facilitating their movement under the applied electric field. This effect may likely become more evanescent upon increasing the temperature, since the interactions between particles and matrix would be weakened.

Table 5 reports the *d*_33_ coefficient of the 60 vol.% PVDF–TrFE composites functionalized with the surfactants “3F-met” and “3Si”, relying on the poling temperature dependence (at 25 °C and 80 °C). Compared with the result of Table 4, an exceptional enhancement was found for the *d*_33_ coefficient, for the samples polarized at both 25 °C and 80 °C, due to the following reasons:

- A higher filler concentration led to a higher piezoelectric response [3,42].
- Being subjected to DC poling instead of AC poling (similar amplitude) led to an improvement of the *d*_33_ coefficient [43].
- A higher poling temperature gave rise to considerably increased *d*_33_ values [39].
- At 80 °C, very few differences in the *d*_33_ coefficient were observed between the surfactant B and C. At 25 °C, on the other hand, a twofold difference was obtained, and the piezoelectric response was higher in the case of the functionalized particle C, which correlated with the explanations of the above result.

It is noteworthy that the *d*_33_ values for our samples polarized at 80 °C were higher with respect to those reported in the literature (i.e., 5 pC/N) [33]. Indeed, the composites in [33] were poled at a higher temperature (120 °C) under 5 kV/mm for a longer time (30 min). Furthermore, they were subjected to hot pressure (at 25 MPa and 120 °C), which would decrease the porosity so as to improve the dielectric and piezoelectric properties. In our case, we treated the samples with a higher poling amplitude (~10 kV/mm), but for a shorter amount of time (20 min, versus 30 min in [33]). A higher poling field speeds up the polarization process; conversely, if polarization occurs during a fixed amount of time, a higher poling field leads to a higher value of the *d*_33_ coefficient [44]. Furthermore, in our work, we poled the samples at a lower temperature (80 °C), which was far from to the Curie temperature of BaTiO_3_ (~120 °C) [13]. Consequently, the condition of polarization (i.e., temperature, time, amplitude) strongly influenced the piezoelectricity. Another factor to be taken into account is that the samples that we employed were considerably thicker than those in Li’s work (19 μm versus 1.3 μm), which led to a higher *d*_33_ coefficient [28]. Last but not least, the geometry of the capacitor that was tested in Li’s work may have differed from ours; as reported in Section 3.6., the ratio between *A_F_* (the area on which the force is applied) and *A_C_* (the area of the electrode) may be dissimilar, which in turn may lead to different results.

## 5. Conclusions

The present paper analyzed the impact of the polymer matrix and surfactant on the dispersion homogeneity, rheology, and dielectric and piezoelectric properties of BaTiO_3_/PVDF composites fabricated through the screen-printing technology. 

Samples were elaborated with two types of polymers: PVDF–HFP (non-ferroelectric) and PVDF–TrFE (ferroelectric), functionalized with three different surfactants including two fluoro-benzoic (“3F-ben” and “3F-met”) acids and a fluoro-silane (“3Si”). The results were compared with composites without surfactant.

FTIR spectra of the functionalized BaTiO_3_ particles revealed that the surfactant “3Si” degraded and/or evaporated during the functionalization process, while “3F-ben” and “3F-met” established strong interactions with the BaTiO_3_ surface.

Rheological tests revealed that the PVDF–TrFE inks were characterized by higher viscosity with respect to those with PVDF–HFP, and that the presence of the surfactant did not significantly affect the viscosity itself.

Profilometry analysis revealed that the roughness of PVDF–TrFE composites was lower, implying that the interface between PVDF–HFP and BaTiO_3_ was less energetically stable.

SEM cross-section micrographs of the composites made it possible to conclude that, regardless of the surfactant, the compatibility between the PVDF–HFP matrix and the filler could not be enhanced, due to these composites demonstrating a certain number of cavities at the interface between the two phases. However, “3F-ben” and “3F-met” positively impacted the homogeneity of the filler dispersion in the PVDF–TrFE composites and stabilized the interface between the two particles, while “3Si” did not give rise to any significant improvement.

Regarding the dielectric properties, neither surfactant nor matrix seemed to notably affect the loss tangent. With respect to the composites with 30% BaTiO_3_ in PVDF–TrFE, the surfactants “3F-ben” and “3F-met”, which enabled a better homogeneity of dispersion, exhibited a higher relative permittivity ($\varepsilon_{33}^{\prime }$) as opposed to the “3Si” and “None” formulations. The PVDF–TrFE composites seemed to have a higher permittivity, with respect to their PVDF–HFP counterparts, and the choice of the surfactant did not affect the permittivity in the PVDF–HFP composites. In composites with 60% BaTiO_3_, no significant impact of the surfactant was observed, but their permittivity was revealed to be higher (especially at higher poling temperatures).

The PVDF–TrFE matrix gave rise to a larger piezoelectric charge coefficient (*d*_33_), as compared with the composites with a PVDF–HFP matrix. Since PVDF–TrFE is a ferroelectric polymer, it exhibits better electric field transmission than its non-ferroelectric counterpart. Another probable reason was the particle debonding phenomenon observed through the SEM micrographs. Composites with the surfactants “3F-ben” and “3F-met” were characterized by slightly lower *d*_33_, because those without surfactant or with the surfactant “3Si” showed some anisotropy in the particle distribution along the poling direction. Moreover, the presence of strong interacting surfactants might have hindered the dipole orientation at the interface between the two phases. Lastly, in addition to the effect of the surfactant and the polymeric matrix, the filler concentration and the poling temperature were also demonstrated to have a strong impact on the material properties. A high filler concentration (60% versus 30%) and an elevated poling temperature (80 °C instead of 25 °C) made it possible to substantially enhance the dielectric and piezoelectric responses.

Future perspectives of this experimental work will focus on material and process improvements, as well as on further analyses aimed at the following:

- Performing plasma fluorinated functionalization of the PEDOT layer in order to improve the wettability of PVDF on the electrode; this is likely to reduce the roughness and the waviness of the composite layer [45] and, thus, not only improve the performances of the composites, but also reduce the uncertainty of the thickness (which makes it hard to carry out consistent comparisons between each formulation, in terms of permittivity and loss tangent).
- Adding other surfactants (e.g., dopamine dichloride) to enhance the homogeneity of the dispersion and reduce the phase separation between triethyl phosphate (TEP) and PVDF, preventing segregation during screen-printing and ink storage [23].
- Carrying out hot pressure on the composite layer to reduce porosity [33].
- Performing BaTiO_3_ particle calcination at high temperature [33].
- Using other matrices (epoxy, PU, PLLA) [46,47] or ceramics (KNN, BZT-BCT) [48,49], and comparing the results.
- Testing other solvents (DMAc, DMF, etc.), which show higher solubility with PVDF [50].
- Carrying out AFM to provide a map of the ferroelectric domains and imaging of the dipole distribution in the material [51], in order to give a more solid explanation of the relationship between the effect of each surfactant and the value of the *d*_33_ coefficient.
- Performing XRD to assess the variation in crystallinity of PVDF [52], due to the lattice distortion induced by the ceramic inclusions [53]. Furthermore, a variation of the β-phase content could be induced by the solvent evaporation process; a raise in temperature, which implies an increment in the solvent evaporation rate, would provoke a decrease in the β-phase percentage [54]. Another future development may be to investigate whether/how the presence of the surfactant affects the crystallinity.

## Figures and Tables

**Table 1:** Chemical and physical properties of PVDF–HFP and PVDF–TrFE (as declared by the manufacturer).

	PVDF–HFP	PVDF–TrFE
**Chemical** **formula**	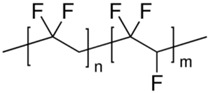	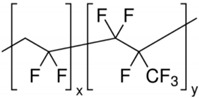
**Composition**	80% PVDF–20% HFP	80% PVDF–20% TrFE
**Density**	1.78 g/mL	1.78 g/mL
**M_w_**	~455,000 g/mol	~420,000 g/mol
**Dielectric permittivity**	10 (@ 100 Hz)	12 (@ 1000 Hz)
**Shape**	Pellets	Powder

**Table 2:** Chemical and physical properties of the surfactants (as declared by the manufacturer).

	Name	Formula	M_w_	Info
**3F-ben**	2,4,5-Trifluorobenzoic acid	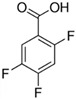	176.09 g/mol	Solid powder T_m_ = 34–36 °C
**3F-met**	3-Fluoro-4- (trifluoromethyl)benzoic acid	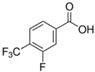	208.11 g/mol	Solid powder T_m_ = 174–179 °C
**3Si**	Trimethyl(trifluoromethyl)silane	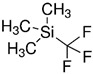	142.19 g/mol	0.5 mol/l, in THF T_b_ = 40 °C
**None**	(No surfactant)	-	-	-

**Table 3:** Average roughness (R_a_) and average thickness (t_a_) of composites with (a) 30% BaTiO_3_ in PVDF–HFP, (b) 30% BaTiO_3_ in PVDF–TrFE, and (c) 60% BaTiO_3_ in PVDF–TrFE.

	3F-ben			3F-met			3Si			None		
	(a)	(b)	(c)	(a)	(b)	(c)	(a)	(b)	(c)	(a)	(b)	(c)
**R_a_ (µm)**	1.2	0.4	-	0.9	0.2	1.2	0.5	0.2	1.5	0.6	0.3	-
**t_a_ (µm)**	7.2	5.5	-	6.4	6.2	18.5	6.5	6.3	19.3	7.1	7.3	-

**Table 4:** Piezoelectric coefficient (*d*_33_) of PVDF/HFP and PVDF/TrFE composites (30% BaTiO_3_).

*d*_33_ (pC/N)	3F-ben	3F-met	3Si	None
**PVDF–TrFE**	0.86	0.86	2.02	1.58
**PVDF–HFP**	0.09	0.12	0.72	0.43

**Table 5:** The *d*_33_coeffcient of PVDF–TrFE composites (60% BaTiO_3_, surfactants “3F-met” and “3Si”).

*d*_33_ (pC/N)	3F-met	3Si
**25 °C**	1.61	3.02
**80 °C**	7.03	7.90

## Data Availability

All data generated or analyzed during this study are included in this published article.
